# What Is the Attitude of Romanian Smallholders Towards a Ground Mole Infestation? A Study Using Topic Modelling and Sentiment Analysis on Social Media and Blog Discussions

**DOI:** 10.3390/ani14243611

**Published:** 2024-12-14

**Authors:** Alina Delia Călin, Adriana Mihaela Coroiu

**Affiliations:** Department of Computer Science, Babeş-Bolyai University, 1 M. Kogălniceanu Street, 400084 Cluj-Napoca, Romania

**Keywords:** ground mole, sentiment analysis, NLP, human mole relationship, environment, pesticides, blind mole rat, small farmers, smallholders, topic modelling

## Abstract

In this paper, we analyse the attitudes and sentiments of Romanian smallholders towards mole infestations, as expressed in online contexts. A corpus of texts on the topic of ground moles and how to get rid of them was collected from social media and blog thread discussions. The texts were analysed using topic modelling, clustering, and sentiment analysis, revealing both negative and positive sentiments and attitudes. The methods used by farmers when dealing with ground moles involve both eco-friendly repellent solutions and toxic substances and pesticides. Even well-intentioned farmers are discouraged by crop and lawn damage, resorting to environmentally aggressive solutions. The study shows that the relationship between humans and moles could be improved by active education on effective ecological agricultural approaches.

## Simple Summary

This study examines the attitude of Romanian small farmers towards ground mole infestations, analysing texts from social media and blog discussions. We explore the concerns and attitudes of these small farmers, and what methods they use to repel moles, with a focus on environmental impact and relevant aspects for the human-mole relationship.

## 1. Introduction

In Romania, the European Mole (*Talpa europaea*) is a mammal that belongs to the Talpidae family, lives primarily underground, and is considered a pest of farmland and lawns [1]. However, economic assessments suggest that farmers may be more likely to be annoyed by the presence of moles than to actually suffer significant damage, with the cost of mole control higher than the cost of mole damage [1,2]. Although the molehills they build can pose some problems for farmers, these animals are actually more beneficial to the soil and crops because they feed on other potential pests (insects, larvae, and earthworms). In this research, we aim to study the opinions and attitudes of Romanian small farmers towards ground moles and identify the most utilised types of interventions in dealing with a mole infestation. This is performed using machine learning (ML) methods (such as Natural Language Processing or topic modelling) to analyse online posts from social media and blogs. Below, we present the background and motivation of this study, then formulate four research questions that we focus on in this work.

**Figure 1:**
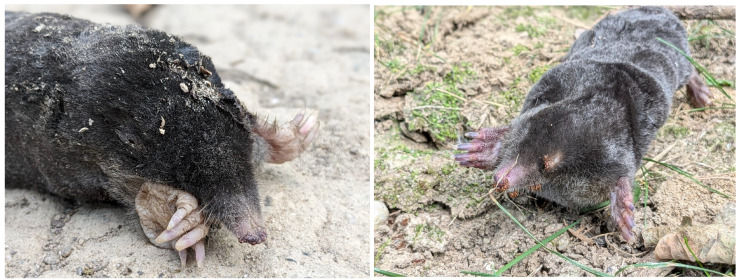
Overground dead mole pictured in Făget Forest, Cluj, Romania, October 2023 (**left**) and in Dumbrava Forest, Sibiu, Romania, August 2024 (**right**). Photo credit Mihai Cuibus.

As a fossorial mammal, the European Mole spends much of its time in vast tunnel systems that can span more than 1000 metres in length and take up to 220 days to build, resulting in strong shoulders, large front paws, and claws that allow efficient digging (see Figure 1) [3]. They are very adapted to this environment and lifestyle: they have poor vision, very acute hearing for low-frequency sounds, using sensitive touch and odour to navigate and defend their territory [2]. Moles breed in spring and live mainly as solitary animals. Mostly foraging for food, they thrive where earthworms are abundant, and their activity (molehills) is in sync with their food [4]. This species is in the Red Book of Endangered Species and is protected by law in several countries. The use of biocides and chemical products in agriculture poses a significant threat to soil invertebrate prey and European Moles, leading to changes in soil quality, such as compaction, erosion, pollution, salinisation, loss of organic matter, biodiversity, floods and landslides, or even climate change [5].

A similar underground activity often results in confusion among Romanian farmers with that of the Eurasian blind mole rat of the Spalacidae family, genus Spalax [6], which has at least three known species in Romania, separated by the Carpathian Mountains: Spalax graecuss, Spalax antiquus, and Spalax istricus [7]. It is also a fossorial species, digging extensive and elaborate tunnel systems with nesting chambers, storerooms, and latrines. As opposed to moles, blind mole rats are slightly larger and feed on bulbs, roots, tubers, and other underground parts of a range of plants [8]. They have eyes under the skin, but no eye openings. Blind mole rats are also protected by law as they are rapidly declining or extinct in some areas due to the expansion of agriculture, the widespread use of deep tillage, and overgrazing [9].

**Figure 2:**
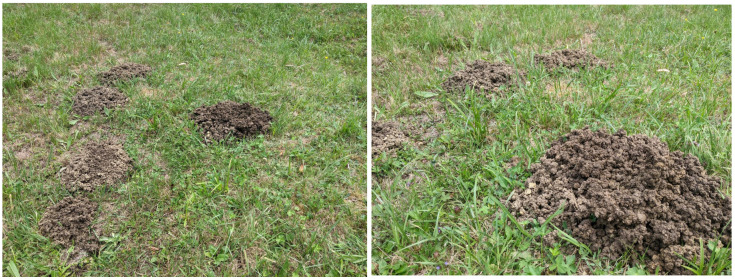
Molehills in Dumbrava Forest, Sibiu, Romania, August 2024.

Another similar animal that is sometimes confused with the mole due to its feeding behaviours is the common shrew (Romanian “Chițcan”—Crocidura leucodon, Sorex araneus, and Sorex alpinus) that feeds on invertebrates (arthropods, earthworms, and snails) but also on vegetative matter [10]. However, this animal lives in various environments, such as dense vegetation, rock cracks, woodland, scrub, road verges, or hedges, sometimes creating holes in the ground or doing crop damage (see Figure 2).

Because ground moles are often confused by smallholders with small mammals of similar activity and crop damage, like blind mole rats or shrews, we extend the aim of this study to include all three species.

It seems that the greatest threat to these animals is their status as pests from the perspective of the agricultural sector, which is currently the greatest threat to ecology [11]. In his book *In Defense of the World’s Most Despised Species: Why we love some species but hate most, and why it matters*, Ernest Small presents significant threats to biodiversity and their impact on humanity, looking at the biased behaviour of humans passionately loving some while passionately hating others [11].

Social media and online blog discussions have been used in the past decades by people as a primary source of quick information, product/experience sharing of the latest innovations, peer advice, and consumer influence [12,13]. As they make it easier for people to share ideas, viewpoints, and knowledge via online networks and communities, social media can also work to counteract and obstruct the spread of false information or bring different perspectives to the table [14] or as a learning tool [15]. On the other hand, social media attacks that spread misinformation can create major damage in a short time, especially in the context of political elections [16,17], being a threat to democracy, or, in the case of health crises such as the COVID-19 pandemic [18], exacerbating public health issues. In this context, digital text analysis methods from the domain of artificial intelligence, specifically Natural Language Processing (NLP), have been developed and researched to process and extract information from this vast amount of content generated online. We can extract the main topics of conversation, namely sentiments expressed by users, and thus better understand their sentiments, opinions, and attitudes related to specific issues.

Some studies [19] explore ML methods for identifying agricultural named entities, using pre-trained BERT (BERT stands for Bidirectional Encoder Representations from Transformers) for information extraction techniques. By integrating the textual attributes of agricultural entities, a collaborative multi-feature model (PBERT-BILSTM-CRF) is created, providing a robust scientific basis for agricultural knowledge graphs and Q&A systems.

Gokcimen et Das [20] used BERTopic and LDA (LDA—Latent Dirichlet allocation) on a collection of texts pertaining to climate change, from multiple blogs and social media platforms, to determine the public’s insights and attitudes towards climate change. The main topics are identified, as well as the similarities between them, highlighting the importance of online platforms to shape the public’s opinions.

Jiang et al. [21] categorise agricultural documents according to natural hazards using BERT multilingual and CamemBERT models, extensions of the BERT model. Their prediction test validated the generalisability of a fine-tuned BERT-based model for topic prediction of French Plants Health Bulletins in the plant health domain.

The BERT-based fine-tuning classification model, created by Yang et al., specifically targets the subtle variations in semantic information and user questions within agricultural question-answering systems. Contrasting with models such as Bi-LSTM (Bidirectional Long Short-Term Memory), Transformer, and BERT-based fine-tuning, the BERT-based model demonstrated good levels of accuracy, precision, recall, and weighted harmonic mean of accuracy and recall. The proposed system demonstrated high precision on the Common Crop Disease Question Dataset, due to its simplicity, few parameters, and rapid processing speed. Consequently, it is a highly efficient option for handling common crop disease questions [22].

Chen et al. [23] present a BERT Pre-training Approach model and Conditional Random Field for the AgriRE dataset, which is a dataset documenting annotated agricultural text entity relationships. The model extracts entity relations from agricultural text features. Empirical investigations conducted on the AgriRE dataset revealed that the AgriBERT model attained the greatest F1-Score (a measure of predictive performance in machine learning, calculated based on precision and recall, with the best value being 100% [24]) of 97.37%, thereby showcasing its exceptional efficacy in the combined extraction of entities and relationships within the agricultural field.

Other papers [25] examine distinct models and their categorisations, with a specific emphasis on six criteria for thorough evaluation: modelling quality, interpretability, stability, efficiency, and additional aspects. The interpretability of topic modelling makes it valuable in several research domains. This paper assesses seven subject models using recommended metrics and proposes four criteria for selecting a model. The authors present a succinct summary of five normative arguments, which encompass theory formulation, testing, study design, dependability, and validity evaluations.

Given the promising results of related work, the present study researches the social media discussions of small farmers and smallholders in relation to ground mole infestations (including similar rodents such as blind mole rats or shrews, which are sometimes mistaken for ground moles). For this purpose, we will use Natural Langues Processing (NLP) techniques such as topic modelling, clustering and sentiment polarity analysis to answer several Research Questions (RQs):

- RQ1. What are the sentiments of Romanian smallholders towards ground moles in public online social media and blog discussions?
- RQ2. What is the attitude expressed online by Romanian smallholders when faced with a ground mole infestation?
- RQ3. Which are the most used domestic approaches for dealing with a mole infestation by Romanian smallholders, based on public online posts?
- RQ4. What types of pesticides are often used by Romanian smallholders against ground moles, based on public online posts?

## 2. Materials and Methods

In this section, we present the methodology used in this investigation to address our research questions presented in the introduction. The first step involved the collection of a dataset that contains text posts on social media and blogs related to moles in the Romanian language.

The dataset was cleaned, pre-processed, analysed, and translated into English as well. Next, we employed Natural Language Processing models for the purpose of extracting information using topic modelling (to cluster and classify the text posts into similar groups and identify the most used approaches for dealing with mole infestations—relevant in RQ2 and RQ3) and sentiment analysis (to identify the sentiments of the users related to this topic present in their online posts—relevant for RQ1).

### 2.1. Dataset

#### 2.1.1. Data Collection and Preprocessing

For the purpose of our research, we collected a dataset of social media and blog posts related to moles. We searched the online tools that are generally known to be most utilised by Romanian users for text posts in order to obtain the opinions, attitsude, and sentiments of private individuals towards a mole infestation in their yard, garden, or orchard. We used a Google search and Facebook search with the Romanian terms “cartita” (“mole”) and “cum scapi” (“how to avoid/get rid of”), focusing on the first 4 pages of the results. We excluded from searches any product reviews related to mole traps, pesticides, or pest control as our aim is far from assessing these methods but to understand the attitudes and sentiments behind the choice of each individual on how to relate or get rid of moles. This is performed following public opinions, advice, and learnt lessons expressed through posts and stories presented and discussed by users, outside of the bias of commercial product-specific pages.

Inclusion criteria, oriented towards Romanians’ opinions and attitudes:

- Social media (Facebook) public groups related to moles, gardening, and pests, selecting conversations related to moles and mole rats.
- Social media public posts related to moles and mole rats.
- Online forums and discussions on topics such as gardening, moles, and pests (selecting discussions on moles).
- Public discussion posts to private blogs about moles and mole infestation in small households.
- Public discussion posts on online shop websites that reflect public opinions, attitudes, or sentiments towards mole and mole-avoidance methods.

Exclusion criteria, to avoid bias or commercial products:

- Promotional websites and private blog posts related to mole or pests products like traps or pesticides.
- Promotional social media posts.
- Online shop websites with product descriptions.
- Product reviews on mole traps, pesticides, and other similar products on shopping websites are also excluded.

**Figure 3:**
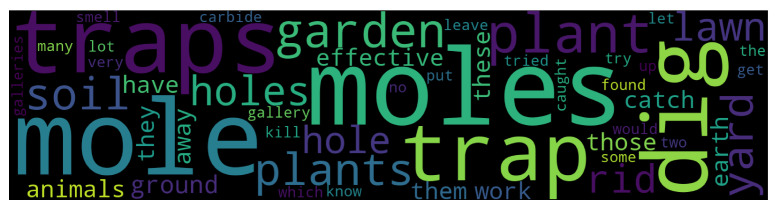
Wordcloud of the most frequent terms.

The posts were extracted from selected online sources (listed in Appendix A, Table A1) in September 2023, to form a collection of texts corpus named CartiteMolesRO. The resulted n=1402 instances of texts were manually processed to correct spelling mistakes and add, where necessary, the specific Romanian language diacritics. The dataset texts were then translated into English using the python Google API *googletrans* library for automatic translation, resulting in the CartiteMolesEN corpus. The collected texts come from blog posts or group discussion topics such as gardening, householding, mole pest infestation, seeds and plants, with Figure 3 presenting a general wordcloud of the dataset with English terms.

**Figure 4:**
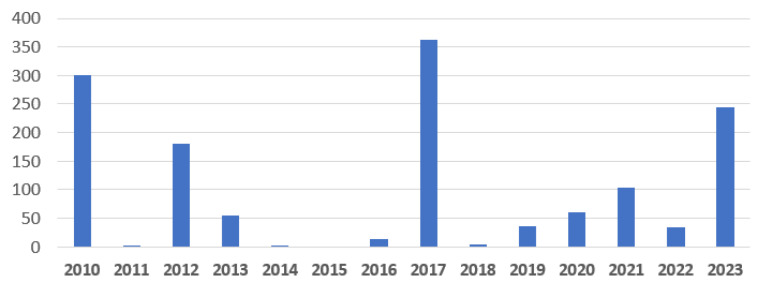
Distribution per year of the dataset posts.

**Figure 5:**
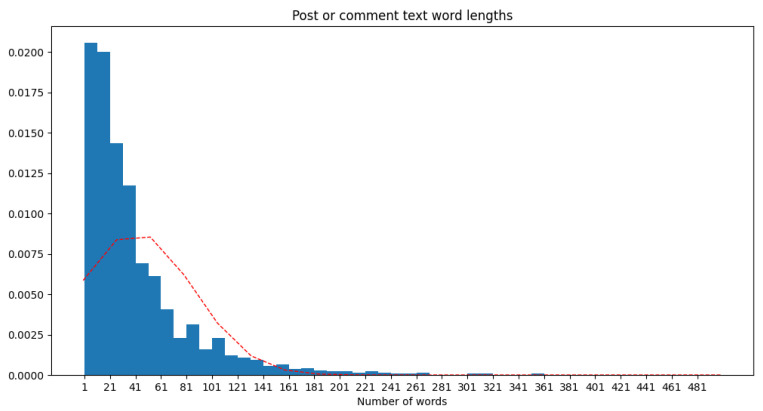
The word length frequency (blue) of the dataset posts and standard distribution curve (red).

Besides the text of the posts, we also collected some metadata, such as year, resulting in a distribution of texts as in Figure 4. From the whole range of 2010 to 2023, we noticed that most posts are from 2010 (301), 2012 (181), 2017 (363), and 2023 (244). The resultant English version of the dataset has the word length statistics per text, as presented in Figure 5. We noticed that most of the included texts fall within a range of 100 words, with some up to 200 words, and only a few are long as over 400 words.

#### 2.1.2. Exploratory Analysis

An initial exploratory analysis of the dataset based on word frequencies is presented in Table 1. Based on the content of the posts, the authors of the online posts are generally believed to be owners of small family farms of 1 ha to 5 ha. They may include one or more of the following: a lawn, a flower garden, a vegetable garden, several crops, or an orchard, generally for their own use.

As mentioned in the introduction, the ground mole is often confused with similar small animals, such as the blind mole rat or the shrew. The data analysed in this study reveal that many individuals do not distinguish between the species; for example, they might be using the name “ground mole” when the bulb or root damage described is typically performed by a blind mole rat. However, the majority of online posts do refer to ground moles based on the context provided by user, and they are the predominant species. The posts contain a total of 748 mentions of the term ground mole (Romanian “cârtiță”), 75 explicit mentions of the blind mole rat (Romanian “orbete”, for which the automatic English translation utilised provides the term “orb”), 33 mentions of mice, 17 of rats, and 7 of shrews.

The most frequent terms were categorised into 4 main categories: (1) terms related to the space and time environmental context; (2) terms related to moles, their characteristics, and their actions; (3) words referring to attitudes against moles that go as far as killing the animals; and (4) words denoting attitudes against moles that focus on repelling or deterring moles without risking to kill them.

In the first category, the spatial context refers mainly to the garden, ground or soil or earth, yard, or lawn, but with less frequency to vegetables/food/orchard/flowers, suggesting that garden or lawn damage would be the most problematic. The temporal references are often mornings (suggestive of mole activity) and spring, with few mentions of weeks (suggesting long-term problems) or winter. The frequent mention of neighbours suggests that this is not a singular issue or a household, but it is influenced by the attitude of the surrounding neighbourhood.

In the second category, moles are associated with holes and galleries, digging, and tunnels. There are many mentions of rats, mice, or other rodents, suggesting that users consider them together. There seems to be a persistent discussion among posts as to the difference between a European ground mole (in Romanian “cârtiță”, latin “*Talpa europaea* L.”), which feeds mainly on earthworms (Lumbricidae), insects or even mice [26], and the blind mole rat (in Romanian “orbete” or “câinele pământului”, latin “Spalax sp.”, which is a herbivore that feeds mostly on roots and bulbs [27]. The distinction is relevant because they carry out different types of damage in the garden (based on feeding habits and types of underground galleries performed). The common mole is actually helpful as it feeds on other pests such as the cricket Gryllotalpa vulgaris, which destroys crops, but its galleries and molehills (see Figure 2) may disturb some crops and result in some damage. However, the blind mole rat feeds on roots and thus may cause significant crop damage directly.

The third category contains many words aiming to get rid of, trap, or kill the moles. Most methods are based on chemicals (carbide, gas, poison, aluminium phosphate, castor, fumigants, TNT, sulphur, and smoke) and some on less pollutant killing methods (flooding galleries with water, attacking them with a shovel, or using trapping devices).

The fourth category of words represent mainly the attitude of repelling moles using designated plants (lilies that produce a foxy smell like Fritillaria imperialis [28,29], and Euphorbia lathyris [30,31]), unpleasant smells (fish heads, mothballs or naphthalene, dung), underground walls or nets, ultrasound devices, vibration devices (including an improvised stick device fixed in the ground with a beer can on top) or animals that hunt or repel them (cats, dogs, or chickens). There are 33 mentions of the “law” meaning some people are aware these animals are protected by law, so they focus on measures that repel without killing.

### 2.2. Topic Modelling

We employed topic modelling methods to extract pertinent information from our collection of mole-related texts. These are unsupervised Natural Language Processing methods that use word groups to summarise textual input and categorise text [25]. In the context of the growing amount of online social context and research, it serves as a link between social science and big data analytics to reveal the complexity of social phenomena [32], and it serves as a powerful tool for communication researchers and information retrieval by analysing vast texts [33] to identify latent themes in documents.

#### 2.2.1. BERTopic

BERT-based topic modelling encompasses the utilisation of BERT’s pre-trained language model to provide contextualised embeddings for documents. Contrary to conventional models such as Latent Dirichlet Allocation (LDA), which depend on word co-occurrence and distribution patterns, BERT accurately captures the semantic meaning of words in relation to their surrounding context [34]. BERT embeddings are next subjected to dimensionality reduction using methods such as UMAP (Uniform Manifold Approximation and Projection) and clustered using algorithms like HDBSCAN (Hierarchical Density-Based Spatial Clustering of Applications with Noise) to create clusters of texts that are semantically related.

For our experiments, we first removed English stopwords based on the nltk corpus provided by the Python library. Next, we used UMAP embedding with the model’s output to represent the main topics and most frequent words in each topic.

#### 2.2.2. Intertopic Distance Map

The Intertopic Distance Map (IDM) [35] is a graphical representation employed in topic modelling to illustrate the interconnections among subjects within a 2D spatial framework, using t-SNE (t-Distributed Stochastic Neighbor Embedding) or UMAP, to decrease the number of dimensions in the data [36]. The IDM in this study is created by applying the t-SNE algorithm to reduce data dimensionality and construct a visual representation in which closely related topics are situated closer to one another, whereas disparate topics are located farther from each other. The perplexity parameter, which controls the balance between local and global data aspects, is set to 30 for the experiment, following tests with various values (10, 20, 40). The chosen learning rate parameter was 100, the number of iterations was 1000, and the distance metric employed was Euclidean.

### 2.3. Clustering Approaches

#### K-Means

Grouping comparable data points together based on their intrinsic properties is an important strategy in unsupervised machine learning. Clustering is extensively employed to identify patterns, derive significant insights, and structure extensive collections of texts [37], such as in our collected dataset. Converting textual data into numerical forms (like a TF-IDF matrix) enables the K-Means algorithm to effectively categorise similar documents or phrases based on the word frequency and distribution [38].

The K-Means algorithm begins by setting a fixed number of cluster centroids. It then assigns each data point to the nearest centroid and proceeds to update the centroids based on the new groupings of points, until the clusters become clearly defined. The effectiveness of the K-Means algorithm largely depends on choosing the right number of clusters, which can be determined using the Elbow Method [39]. Furthermore, we examine the most prominent words in each cluster, to decipher the primary subjects and assign significant labels. We used the scikit-learn implementation, with the following selected parameters: *n_clusters* determined automatically by the elbow method, *init_value* set to 10 (indicating that the algorithm is executed with 10 centroid initialisations), *max_iter* set to 150 (maximum number of iterations), and *convergence_threshold* as default (1 × 10^−4^), because there are no variations for another threshold.

### 2.4. K-Means++

K-Means++ is an optimised version of the conventional K-Means method. An enhanced initialisation step is employed to choose the initial centroids with greater knowledge, resulting in accelerated convergence and generally yielding superior clustering outcomes [40]. By choosing initial centroids that are widely spaced, K-Means++ minimises the likelihood of obtaining unsatisfactory clustering outcomes and accelerates the process of convergence. In comparison to ordinary K-Means, it is typically more dependable and resilient, frequently achieving superior minima.

### 2.5. Sentiment Analysis

Employing sentiment analysers from Natural Language Processing (NLP) involves classifying texts into positive, negative, or neutral, or extracting emotions present in the texts. These tools are generally either lexicon-based or machine-learning-trained. In this paper, we used the TextBlob, Vader and Flair models to assess the sentiment polarity of the collected dataset texts in English. The Roberta “SamLowe/roberta-base-go_emotions” and Distilbert “bhadresh-savani/distilbert-base-uncased-emotion” pre-trained models were used to extract the predominant emotions of the posts.

TextBlob [41] is a Python text-processing library designed for sentiment analysis, translation, classification, part-of-speech tagging, and noun phrase extraction. Vader [42] is a basic rule-based lexicon that was developed with attention to social-media-type content, comprising word terms and emoticons. They both classify sentiment polarity into negative, positive or neutral, while TextBlob also provides a score of subjectivity. While the previous are both lexicon-based, Flair is an LSTM neural-network-based trained model that uses vector word representations [43], trained on a large corpus of English texts.

We also used two models from the HuggingFace platform (which provides a collection of pre-trained models covering a wide range of languages and fields). The first model, “SamLowe/roberta-base-go_emotions” [44], is trained on Reddit data, which is similar to our type of input, to extract 28 emotions. Thus, it is a multi-label classification model with 28 “probability” float outputs for any given input text. The second model for emotion detection is “bhadresh-savani/distilbert-base-uncased-emotion” [45]. It is trained on twitter data, involving knowledge distillation during the pre-training for the classification of 6 basic emotions: joy, sadness, anger, love, fear, and surprise.

## 3. Results

### 3.1. Topic Modelling

#### 3.1.1. BERTopic

**Figure 6:**
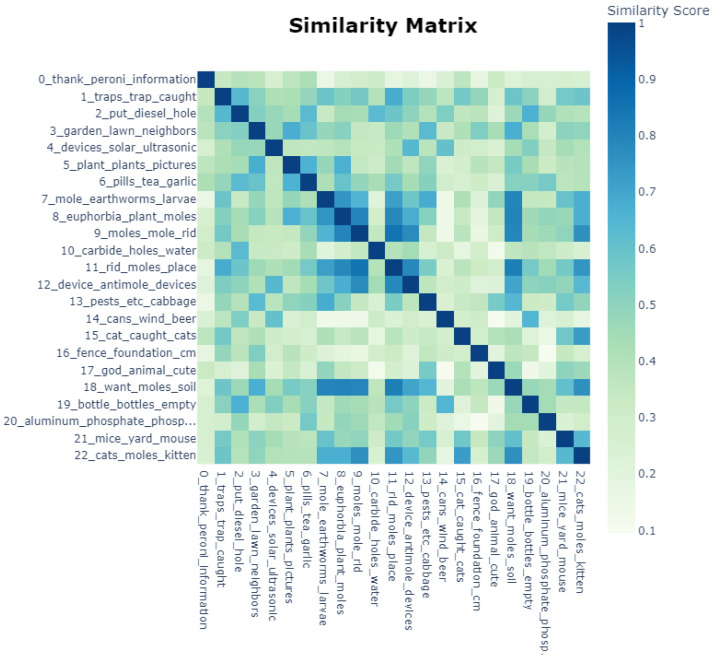
BerTOPIC similarity matrix.

BERTopic identifies 23 clusters of topics from the 1402 posts. Figure 6 presents the topics and the similarities between them.

**Figure 7:**
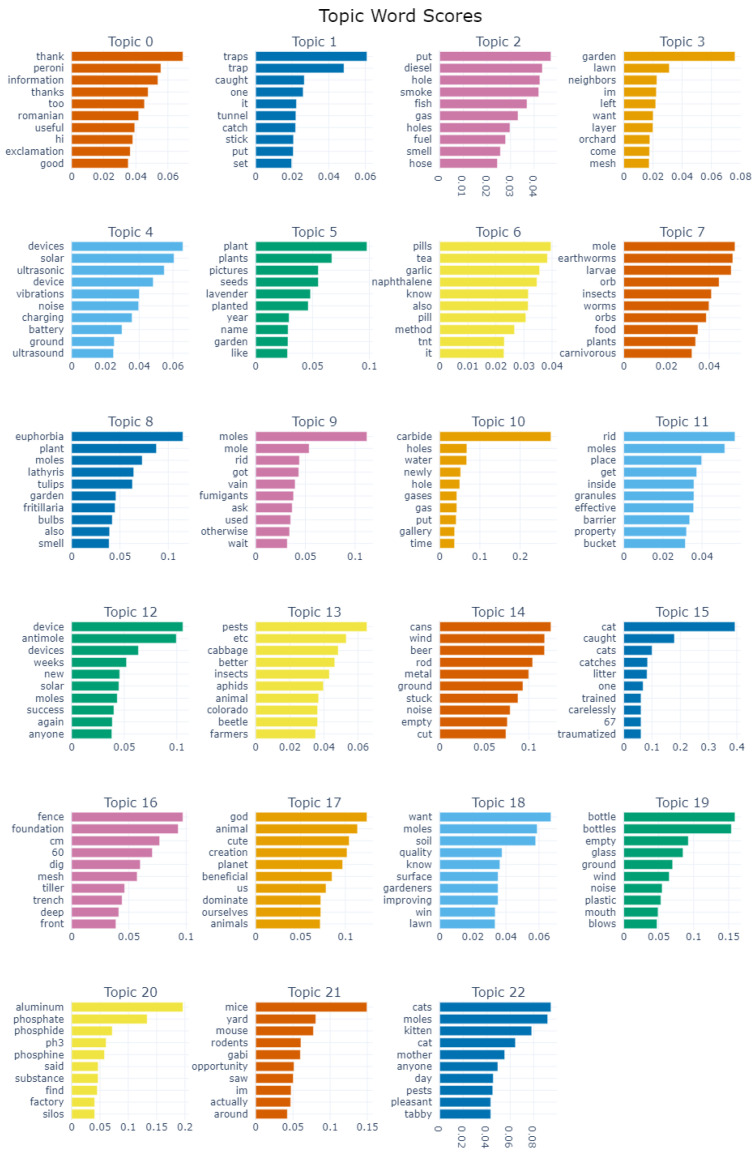
BerTOPIC topics and top frequency words.

Figure 7 distinguishes the top 10 most frequent words in each topic and their frequency. Next, we will analyse these topics and how they relate to each other.

In this sense, we distinguish many topics related to getting rid of moles, as specified by the top keywords in Figure 7. Topics 1, 2, 6, 9, 10, 11 and 12 refer to various trapping or exterminating methods for moles, mostly chemical or aimed at killing them. Topics 0, 14, and 19 focus specifically on the method of using improvised beer cans in a stick to create vibrations in the ground to repel moles, with a more jovial tone. This theme is similar to Topic 4, which includes more designated ultrasound- or vibration-based repellent devices. Topic 20 is related to using aluminium phosphide. Topics 15 and 22 focus on using cats as mole pest control, while Topic 16 discusses deep foundation fences and mesh protection against moles.

Topics 3, 5 and 18 are related to the garden, lawn, orchard, neighbours, flowers, and plants, suggesting the interests of people seeking to protect household surroundings from mole infestation damage. In a similar vein, Topic 8 is focused on using plant-based solutions as repellents for moles.

Topic 13 extends the discussion to other pest problems affecting the garden or crops, including insects (aphids, colorado beetle).

Topics 7 and 21 are quite separate from the others, debating the differences between moles, rats, and mole rats (orbs); their feeding habits; and the impact on the garden and soil.

Topic 17 is also distinguished by the argument that moles are important to the environment, as God-created creatures, cute, and beneficial to the soil or garden.

#### 3.1.2. Intertopic Distance Map

**Figure 8:**
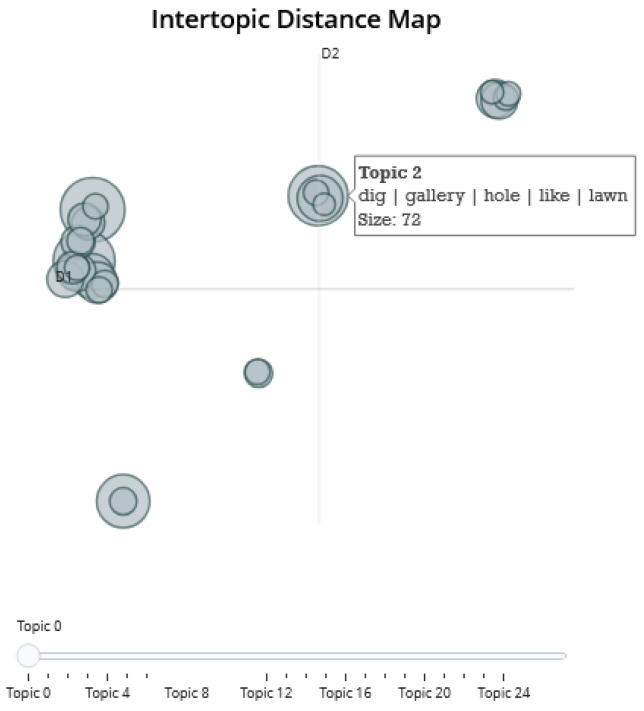
The intertopic distance map.

Using dimensionality-reduction methods, we generate the map by compressing the high-dimensional topic [32,46] representations into a 2D space, resulting in 25 topics in this case. The intertopic Figure 8 enables us to ascertain the degree of similarity or dissimilarity between the fields. The representation offers a comprehensible identifier for each topic, derived from the most indicative terms associated with it. The overlapping topics are similar in nature, and we identify five such groups from the figure.

Group of topics 1: Moles and repellents. This group (0, 3, 6, 13, 11, 18, 23) refers to moles and ways to repel or manage them (mole, hole, dig, anti, rid, repellent, effective, buy, gallery, rid, catch, trap, repel, poison, method, result, work, investment, destroy, damage, affect).

Group of topics 2: Nature and garden. This group (1, 5, 7, 9, 21, 22) includes plants, animals, and natural elements (plant, garden, flower, lawn, bulb, wind, solar, garden, eat, larva, insect, earthworm, animal, dog, live, earth, cat, catch, neighbour, snail).

Group of topics 3 (6, 10, 2, 4, 17): Tools and devices. Words from this group are related to physical tools, substances, and equipment used (trap, catch, wire, anchor, set, stick, push, hole, hose, soak, substance, wind, noise, solar, device, ground).

Group of topics 4: Chemicals and materials. This group (12, 19, 20, 14) focuses on chemicals, materials, and their properties (aluminium, phosphate, grain, phosphine, pill, mothball, carbide, lump, steam, sulphur, cartridge, burn, soak, hose, diesel, smoke, mothballs).

Group of topics 5 (8, 16, 24): Emotions and Interactions. Words from the group are related to emotions and communication related to the topic (good, creation, cute, upset, cool, interesting, laugh, information, thank, useful, wait, reading, exclamation, post, react).

### 3.2. Clustering Approach

#### 3.2.1. K-Means

**Figure 9:**
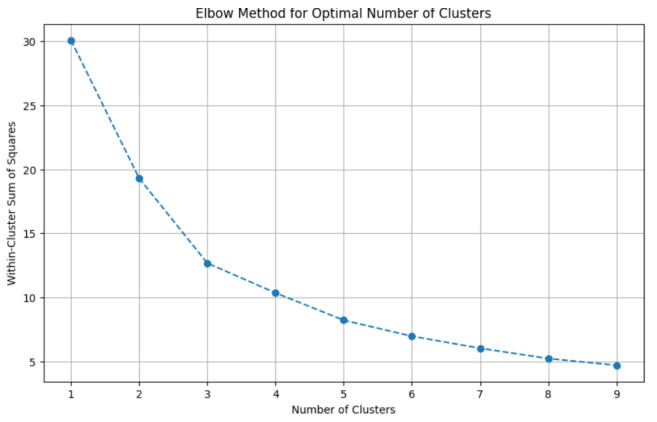
Number of clusters identified automatically using the elbow method.

Figure 9 demonstrates that the ideal number of clusters for our datasets is 3, as emphasised using the Elbow method. The Within-Cluster Sum of Squares (WCSS) measures the total variance within each cluster. It represents how tightly grouped the points are within each cluster. As the number of clusters k increases, WCSS generally decreases because adding more clusters allows each cluster to fit the data more closely [47]. The Elbow Method identifies the point of diminishing returns—the value of k, when the number of clusters is increasing, yields marginal improvements in reducing WCSS. This point often appears as a noticeable “elbow” in the plot of WCSS versus k.

**Figure 10:**
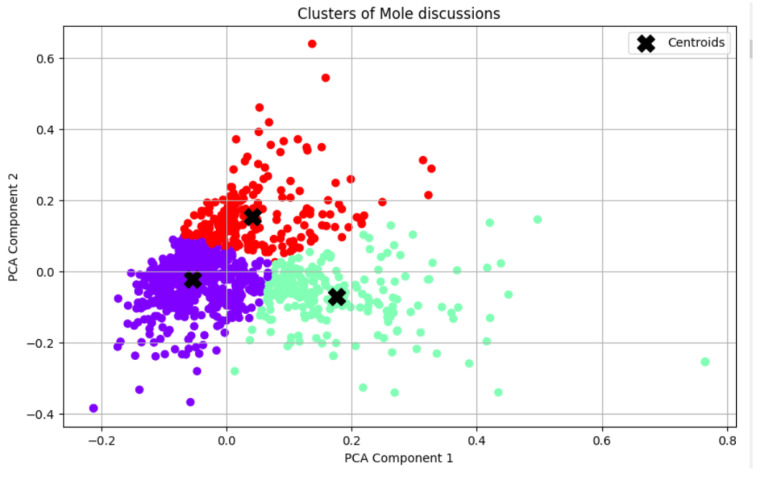
The clusters’ representation: each cluster is represented with one colour, and the centroid is marked with an X in the middle of each cluster.

Following the reduction in the TF-IDF matrix’s dimensionality, we performed K-Means clustering, yielding three clearly defined clusters [48]. We initialised K-Means by instantiating a K-Means object with the specified number of clusters (three in this example) and establishing a random state to ensure reproducibility. Subsequently, we utilised the K-Means model on the reduced TF-IDF dataset and made predictions about the cluster assignments for each individual data point. Ultimately, we represented the clusters visually by graphing the data points, employing various colours to denote their associated clusters, and we displayed the cluster centres (centroids) to enhance the clarity of the representation, as can be seen in Figure 10.

Using the method *Top terms per cluster*, we identified the principal concepts or words that most accurately characterise each cluster [49]. As a working step, we acquired Cluster Centres: we derived the coordinates of the cluster centres (centroids) from the K-Means model that has been trained. Second, we performed Cluster Centres Sort: we employed the argsort function to arrange the cluster centres in ascending order to ascertain the indices of the phrases that exhibit the greatest relevance to each cluster. Then, we performed Retrieve Feature Names: we retrieved the feature names (thematic terms) from the CountVectorizer object. Last, we display the most frequently used terms in each extract and presented the top N phrases with the greatest contribution to the cluster centre for each identified cluster.

We examined these leading terms to pinpoint the main themes or subjects linked to each cluster, therefore facilitating the assignment of significant labels to them. These findings provide good knowledge of the unique features of each cluster and provide useful insights into the empirical data.

Table 2 displays the most frequently occurring words for each cluster obtained from a K-Means clustering analysis. The clusters embody discrete sets of terms that often co-occur in the data, therefore suggesting possible themes or subjects.

Cluster 0 is centred around gardening or agricultural subjects to moles. The inclusion of the terms “mole”, “plant”, and “garden” implies that the data points within this cluster pertain to the interaction between moles and garden plants. This cluster may potentially refer to mole behaviour in gardens or the methods employed to control moles in garden environments.

In Cluster 1 the terms “hole”, “repellent”, and “cat” collectively suggest a recurring motif of deterrents or strategies aimed at preventing moles from excavating or inflicting harm. Discussions under this cluster may encompass a range of mole control techniques, such as the use of repellents or even the recruitment of cats as a natural means of pest management.

The focus of Cluster 2 appears to be on the biology and behaviour displayed by moles. The term “buried” may allude to the subterranean habitat of moles, “reduce” may denote attempts to diminish mole populations or inflict harm, and “blind” emphasises a unique trait of moles, as they are commonly regarded to be functionally blind. This cluster may predominantly emphasise the comprehension of mole behaviour or biology rather than solely focussing on control techniques.

The silhouette score is a quantitative measure employed to assess the efficacy of clustering [50]. The silhouette score is a metric used to evaluate the quality of clustering results in unsupervised learning. It quantifies how well each data point fits within its assigned cluster compared to other clusters. The score ranges from −1 to 1, where higher values indicate better-defined and more cohesive clusters. The silhouette score for our cluster is 0.37, indicating that the items within clusters are quite alike and that the clusters themselves also display considerable resemblance.

**Figure 11:**
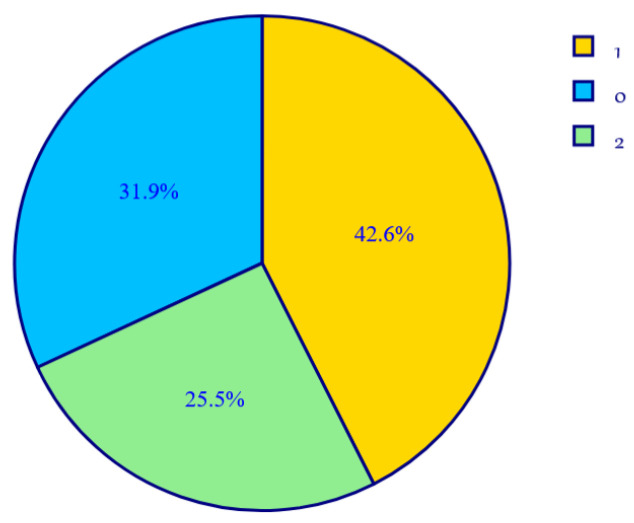
The clusters’ distribution.

The distribution of instances in clusters [51] displays the count of data points allocated to each cluster, therefore offering views into the comparative scales of the clusters. Figure 11 presents identical quantitative data as the proportions of the entire dataset, emphasising the comparative magnitudes of the clusters, suggesting successful data segregation.

#### 3.2.2. K-Means++

**Figure 12:**
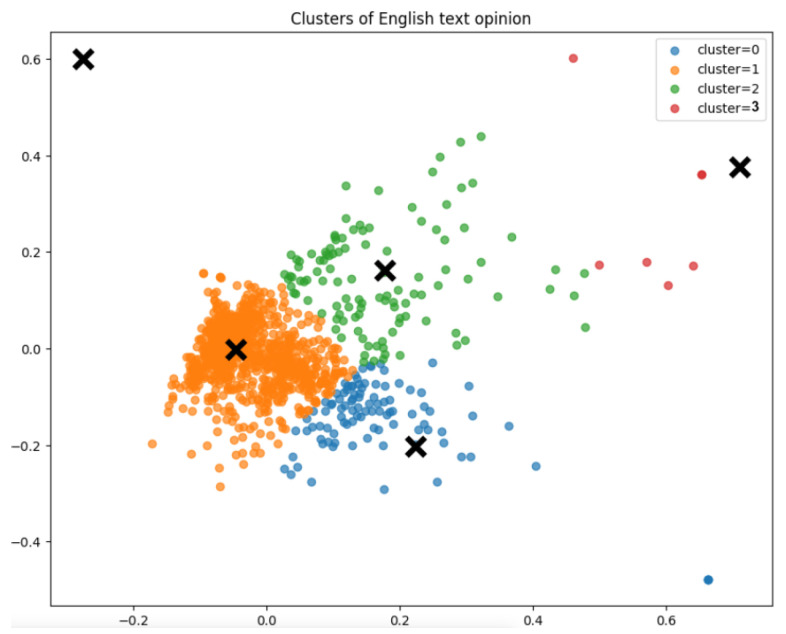
The clusters representation for K-Means++. The centroids of the each cluster is marked with an X.

**Figure 13:**
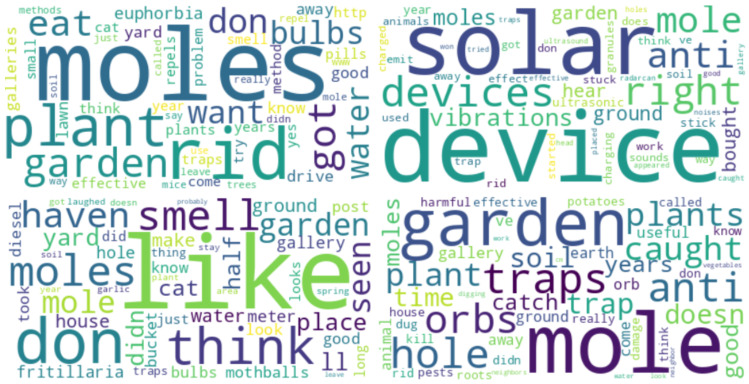
Words frequencies in the clusters.

In the K-Means++ optimisation, we did not pre-determine the value of k. Instead, we allowed the algorithm to detect the potential clusters present in our dataset. The outcome is displayed in Figure 12 and Figure 13. In the context of the K-Means++ algorithm, the value of k represents the number of clusters that the algorithm detects in the dataset. Each cluster is essentially a group of data points that share similar characteristics and are closer to one another based on the chosen distance metric.

### 3.3. Sentiment Analysis

**Figure 14:**
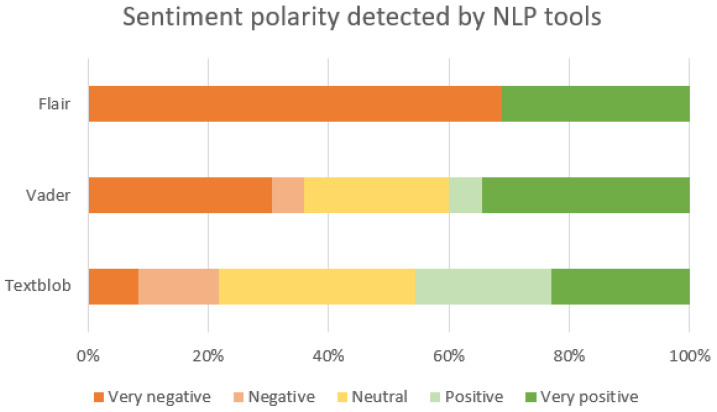
Sentiment polarity with Textblob, Vader, and Flair for the entire dataset.

The results obtained by engaging the tools Texblob, Vader and Flair for detecting sentiment polarity are presented in Figure 14. Each post text in the English dataset is classified given a score in an interval in which extremes are very negative (strong orange colour), very positive (strong green colour), or neutral (yellow coloured). We notice the three tools do not agree on the result: Textblob suggests more positive sentiments (almost 80% neutral or positive), while Flair suggests mostly negative sentiments (70% strong negative). The difference could be marked by a different interpretation of the subjectivity of a sentence (it might express a negative trait but not a personal negative opinion). TextBlob classifies sentences with descriptive or neutral expressions as positive if they lack overt negativity or contain mildly positive language [52]. Flair, on the other hand, appears to be more sensitive to nuanced negativity, potentially interpreting expressions of negative words or critiques as strong negative sentiments, even if they lack a personal opinion [53]. However, we can say that there is a significant amount of strong positivity in these posts, just as there is strong negativity.

**Figure 15:**
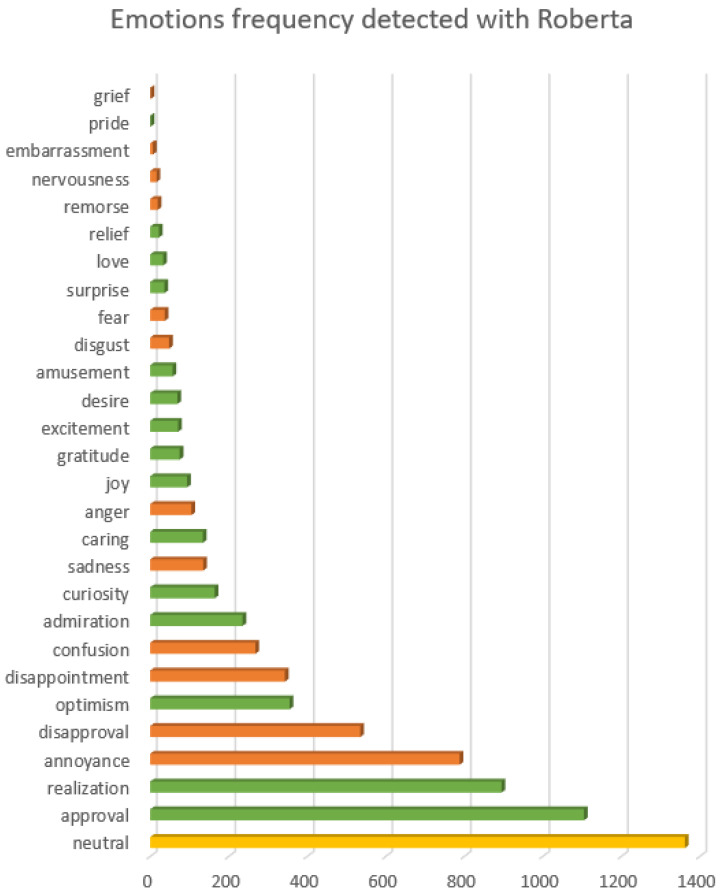
Emotions using Roberta. Colour codes: orange—negative; green—positive; yellow—neutral.

**Figure 16:**
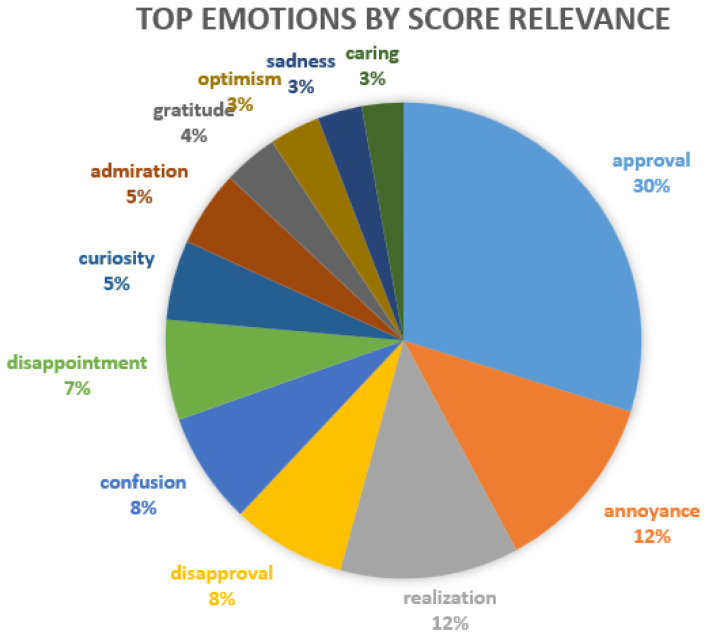
Top-scoring emotions based on Roberta across the dataset.

Thus, to gain more insight into the actual sentiments, we used a pre-trained Roberta model to extract specific emotions found in each of the dataset texts. In Figure 15 and Figure 16, we have a histogram for each emotion and how many of the texts present this emotion in the top five emotions extracted by the model. We notice in this case that positive emotions are predominant and balance the negative ones. The most frequent case is neutrality, as many posts contain sentences that do not express personal opinions, but information and facts, which are objective.

The most frequent positive emotions are approval (perhaps a lot of agreement between methods that do or do not work in repelling moles or sympathy/agreement regarding damage performed by moles and its impact), realisation (these posts express a new situation for many people who ask for advice), optimism, admiration, and curiosity. There is less caring, joy, gratitude, excitement, or love. The most frequent negative emotions are annoyance (mostly at the damage performed and the persistence of moles), followed by disapproval, disappointment, and confusion, with less frequent negative emotions being sadness, anger, disgust, fear or remorse.

**Figure 17:**
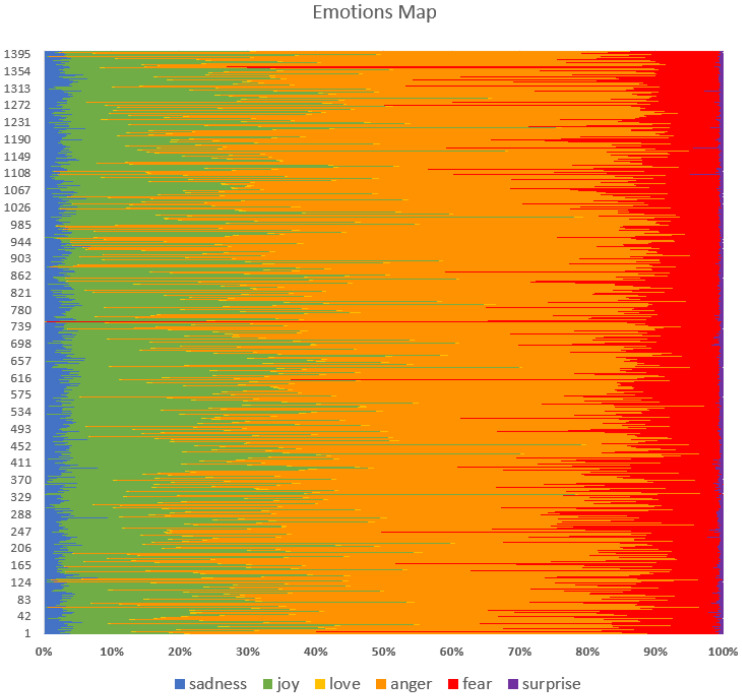
Emotion map using Distilbert for each of the 1402 posts.

**Figure 18:**
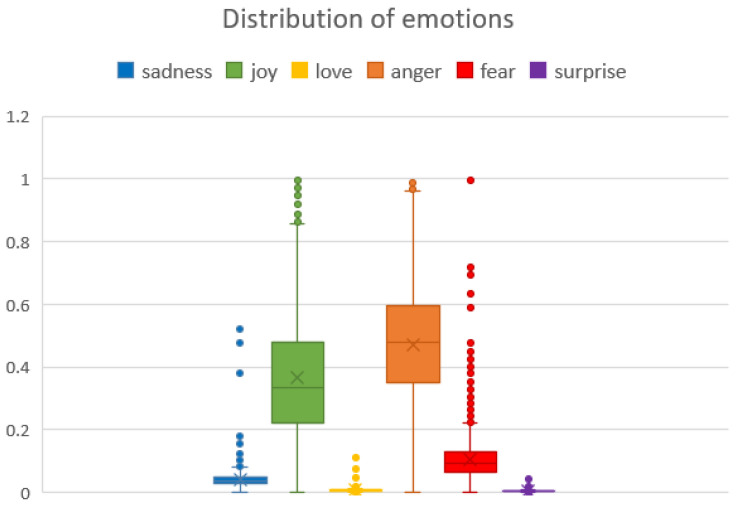
Emotion distribution based on Distilbert across the dataset.

A further analysis of the six basic human emotions (sadness, joy, love, anger, fear, and surprise) was performed using the pre-trained Distilbert model. The results are shown in Figure 17 and Figure 18.

The figures show that the predominant strong emotion is anger, which seems to be specific to the annoyance at the disturbance and damage generated by moles and their persistence in the garden despite various methods used. This is followed by quite strong joy, which can be explained by the fact that many posts have a humorous and jovial tone to the whole situation of fighting a mole infestation and how the methods and devices used are being overcome by intelligent moles. Next is fear, which is less frequent, which can be related to the worries regarding the possible loss of significant investments in crops, lawns, or gardening. The least frequent are sadness, surprise and love.

## 4. Results, Analysis, and Discussion

In this section, we will focus on answering the RQs formulated in Introduction, based on the results obtained using topic modelling, clustering and sentiment analysis approaches.

### 4.1. RQ1. What Are the Sentiments of Romanian Smallholders Towards Ground Moles in Public Online Social Media and Blog Discussions?

As presented in the exploratory analysis and results sections, there is a general tendency to deter moles from people’s loved gardens, lawns or yards. In Table 1, we see among the mole-related words mostly negative terms which associate them with destruction (digging, damage, tunnel, holes), leading to a hostile opinion that they are pests worth destroying. There are fewer positive terms correlated to an attitude of protection, aiming to repel them without killing or hurting, as they are important mammals with their own role in nature and the environment (cute, God, nature, protected, mammal). The BERTopic results in Figure 7 show a similar trend, with only one topic (number 17) considering moles as beneficial for the soil and garden, and the other 19 topics focused on eliminating them by various means.

In terms of the sentiments expressed by people in the online environment, there is a balance between positive and negative emotions. However, the subject of these emotions can vary; for example, there might be negative hatred towards moles, but positive gratitude towards a solution provided by someone to get rid of moles, both in the same post. As such, we can only evaluate at this point the emotions expressed in general in the online discussions about mole infestations. In this sense, Figure 15, Figure 16 and Figure 17 supported by group 5 of topics from the intertopic distance map, show a balanced set of emotions in which the negative ones are anger, annoyance, disappointment, confusion, fear, sadness and disapproval, while the positive ones are approval, realisation, admiration, curiosity, joy, gratitude and optimism. In terms of interpreting these emotions, looking at some posts, one might argue that some people enjoy the challenge posed by moles in their garden or simply like to make fun of their own failure to repel them with certain methods tried. While they might be afraid they will destroy their crops or lawns and disapprove of the moles, they would also be impressed by the animal’s ability to dig large networks of tunnels and survive despite being hindered and being so small. This dynamic of emotions, even though it is mostly oriented against moles, shows that there is still great positivity in people and room for persuasion in favour of moles. We can see from the analysis of the collected dataset (in Table 2 and Figure 13) that the way most people feel about animal species is influenced by how they perceive its impact on the environment.

Thus, it seems that most people are inclined to have a bad opinion on moles, as being nothing more than destructive pests, and are happy to get rid of them. This is in agreement with related work in [1] stating this as a cultural fact and sentiment preference. Economic assessments show that the costs of pest control are higher than the costs of crop damage, suggesting that sentiments drive the actions of people rather than rationality. However, there is a representative online voice in our analysed dataset and also in literature [11] aiming to educate people towards a better sentiment and opinion about moles by emphasising their beneficial role in nature (soil aeration and other pest management) and mammalian cuteness. Based on the ability of people to be impressed, curious, caring and challenged, it would be possible to develop a more positive general attitude towards moles, resulting in less pesticide use and better environmental and human health. Moles and blind mole rats, being subterranean mammals, are well acknowledged for their ecological significance in soil aeration and pest management, which might cultivate a favourable attitude among environmentally informed individuals or those engaged in agriculture. Nevertheless, there may also be a prevailing unfavourable attitude, especially among certain groups that view these animals as troublemakers that harm crops and gardens.

### 4.2. RQ2. What Is the Attitude Expressed Online by Romanian Smallholders When Faced with a Ground Mole Infestation?

We identify three main attitudes from the topic modelling in Figure 7. One is tolerance towards moles and being committed to living in harmony with them, considering that they have their specific role in the environment and the small damage is irrelevant. The second is against moles, in which people do not welcome their presence, but they are orientated towards ecological repellent methods, such as having a cat or using ultrasound devices. These first two categories are generally aware of animal protection laws. The third main category is against moles and involves people being ready to employ killing methods and toxic pesticides such as phosphides, ignoring any law for the protection of animals and the environment.

Moles are usually seen badly by smallholders and farmers in Romania, mostly because they damage crops and gardens (see Figure 14). The negativity can occur because the moles destroy crops, dig holes, and disturb roots or soil, causing farmers to lose food or money invested in the garden or lawn. The result is a determined attitude to get rid of them, first by mild methods, using handy solutions. If they do not work, some accept them and the disturbances they make. Others use more aggressive methods to kill them and keep them away. The attitude is also evident from the keywords indicated in Figure 13. Considering the Figure 16, we may observe certain responses focused on prompt mitigation and positive control strategies.

The only positive attitude is linked to the desire to be environmentally friendly and to live an integral life in deep connection with nature. However, while this good intent is present in many people, they do not possess the knowledge and abilities to integrate specific, cost-effective, and eco-friendly methods. The result is that they are discouraged by the crop and financial losses from the damages caused by moles or blind mole rats to the farm in which they have invested time and money. As a result, they give up easily, first resorting to mild and then to more aggressive pest control measures.

### 4.3. RQ3. Which Are the Most Used Domestic Approaches for Dealing With a Mole Infestation by Romanian Smallholders, Based On Public Online Posts?

The first line of domestic approaches to deterring moles are mostly based on creating unpleasant conditions for the hearing and olfactory senses of the mole by creating vibrations or bad smells. These are generally also the cheapest solutions and allow improvisations.

The identified topics and clusters (Figure 7 and groups of topics 1 and 4 from the intertopic distance map) show various methods utilised in order to control mole populations.

Environmentally friendly methods include the possibility of using natural predators such as cats or dogs or ultrasonic devices, vibrations, and noise. A popular and cost-effective home-made solution involves placing a stick in beer cans in the gardens to produce sound vibrations. Barrier-like methods are also used, namely a deep foundation fence and mesh protection on the lawn or garden area to be protected. The most eco-friendly solution, which is also cost-effective, uses plants such as lilies that produce a foxy smell, like Fritillaria imperialis or Euphorbia lathyris. Other smell-repellent-based methods involve introducing rotting fish heads, mothballs/naphthalene, or dung in the molehills or galleries.

The second line of intervention is based on chemical substances and traps that kill the animal underground: carbide, gas, poison, aluminium phosphate, castor, fumigants, TNT, sulphur, and smoke. They are introduced at the end of a tunnel and then covered to keep the substance’s steam underground, reaching through the air in the galleries to the targeted mole. Similar methods involve flooding mole galleries with water.

The old-fashioned way of catching moles is less popular; it involves waiting for them to dig in the morning to attack them with a shovel or using mechanical trapping devices. Another less used but recently developed method is live the trapping and trans-locating of the animal, which is considered to be eco-friendly. However, it seems to be problematic for survival reasons (food sources, availability of territory).

The first line of intervention is generally mild towards the environment and animals and also involves low-cost and handy solutions. However, they do not always work. Popularising eco-friendly and cost-effective solutions and the correct ways to use them in the case of a mole infestation can be very helpful in preventing the second line of intervention, which is more aggressive towards both moles and the environment. Also, educating farmers on the differences between moles and blind mole rats and how to deal with each of them is very important to ensure effectiveness as they respond to different interventions.

### 4.4. RQ4. What Types of Pesticides Are Often Used by Romanian Smallholders Against Ground Moles, Based On Public Online Posts? 

A very commonly used pesticide (identified in Topic 20 of Figure 7 and group of topics 4 from the intertopic distance map) is aluminium phosphide (AlP), also known as phosphide. It is commonly used in Europe for mole pest control, and it kills the animal by depressing the nervous system and its respiratory function [54]. It is a highly toxic substance for humans and a significant water pollutant. Other chemical substances used are naphthalene, which is now considered a carcinogen, and carbide, which can cause throat irritation, coughing, and difficulty breathing, being a significant contributor to the greenhouse effect and environmental degradation.

### 4.5. Ethical Considerations

The corpus collected in this study contains publicly available online information. Personally identifiable information such as names or usernames have been removed from the texts.

The dataset collected is focused on topics related to getting rid of moles and is biased towards active action on mitigating a mole infestation. Considering that we do not have enough information on the demographics of people posting and the texts are online, the dataset is perhaps biased to include only digital users. Considering that the main subject of the blogs refers to getting rid of moles, the population living in harmony with moles is under-represented in the dataset, but there are still many positive opinions expressed. This is because most blog threads and posts focus on the topic of repelling moles, which involves proactively searching for solutions, while people who live happily with moles in their gardens do not need to discuss this extensively and proactively online. As such, it is unclear how representative these findings are of the whole (digitally active) population of Romania.

In this study, we discuss both ground moles and blind mole rats, because they are confused by many users, and the posts are often mixed, making it hard to distinguish between them. Future work would require identifying the explicit (name used in the text) or implicit (type of damage and traces left by the animal as explained in the post) references to each of the two species and separating them. This way, we can have a clearer view, attitude, and opinion in both cases.

## 5. Conclusions

This study uses NLP techniques, in particular topic modelling, sentiment polarity, and clustering approaches to analyse the public online opinion of Romanian smallholders towards ground mole infestations. For this purpose, we collected a corpus of online posts related to ground moles from social media and blogs and analysed the texts, focusing on four main research questions.

Romanian smallholders’ sentiments towards ground moles (RQ1) are mostly negative, as they view them as harmful pests, linking them to the losses in crops. Farmers feel mostly annoyed and worried, as revealed by the analysis of emotion polarity. However, there is a smaller percentage of people who hold a more balanced and positive opinion, acknowledging moles’ ecological advantages. According to the online discussion, when dealing with a mole infestation (RQ2), most Romanian smallholders are keen to solve the issue and limit damage by any means, while very few draw attention to the potential beneficial effects of mole activity. While most farmers aim to kill the animals, some show interest in non-lethal, eco-friendly alternatives, suggesting that they may be receptive to more sustainable methods. The investigation revealed a wide variety of strategies used by farmers to control mole infestations (RQ3): employing chemical compounds and pesticides, traps, repellent plants, domesticated predators (cats or dogs), repellent ultrasound devices, or home improvised repellent installations. The most common pesticides and chemicals used to combat moles (RQ4) are aluminium phosphide, naphthalene and carbide.

While there is still a common negative impression of ground moles, the evidence points to an increase in knowledge and enthusiasm for ecological and conservation-focused strategies. This is an opportunity for public awareness initiatives to promote environmentally friendly pest control methods and improve knowledge of moles’ ecological functions. It could be possible to accommodate farmers’ pragmatic concerns and reduce the environmental impact of mole control efforts by promoting a more balanced perspective.

## Figures and Tables

**Table 1:** Most occurring words and their frequency based on category.

Environment Context		Mole Related Words		Kill or Trap Moles		Deter Moles	
garden	216	moles	477	rid	143	plant	95
ground	115	mole	328	water	99	plants	67
soil	110	hole	126	traps	99	smell	53
yard	87	holes	96	try	71	protected	44
year	86	galleries	86	tried	70	planted	44
time	80	gallery	75	method	65	bulbs	43
lawn	75	animals	54	trap	64	stick	43
earth	55	animal	47	catch	60	dog	42
spring	39	eat	47	caught	59	cat	36
house	38	mice	47	carbide	58	net	34
solar	38	area	46	effective	55	law	33
neighbors	37	roots	45	kill	54	wind	32
drive	37	digging	42	anti	51	noise	27
haven	37	tunnels	41	bought	49	ultrasound	27
morning	35	dug	41	gas	49	mothballs	27
days	34	damage	36	pills	48	vibrations	26
potatoes	33	live	35	solution	48	garlic	25
cut	32	small	35	device	44	oil	25
metres	32	tunnel	29	devices	43	cats	24
trees	31	orbs	26	diesel	40	wire	24
food	29	insects	24	methods	36	beer	23
flowers	29	strong	24	sulfur	34	sticks	21
worked	28	mouse	24	hoe	34	bottle	20
help	28	rodents	22	poison	32	ultrasonic	19
neighbor	28	rats	19	pests	31	fish	19
seen	26	species	19	stuck	31	euphorbia	18
pieces	26	rat	18	remove	26	flower	17
day	26	god	18	hose	26	cans	17
summer	26	nature	18	aluminum	25	metal	17
orchard	25	digs	15	destroy	23	current	17
money	25	smells	15	smoke	23	chicken	16
field	23	earthworms	15	toxic	20	fritillaria	16
country	23	grass	15	fight	19	repel	15
winter	22	leeches	15	destroyed	19	smells	15
land	20	seeds	15	bucket	18	concrete	15
stay	20	orb	15	spade	18	repellent	14
hours	20	channels	14	substance	18	mesh	13
surface	20	trenches	14	desperate	17	repels	13
fresh	20	cute	11	castor	17	pet	13
weeks	18	mammals	11	fuel	17	onions	13
fence	18	blind	10	phosphate	15	heads	13
tulips	16	outside	10	fumigants	13	naphthalene	12
vegetables	15	romania	10	killed	12	organic	12
vegetable	14	buried	10	die	12	dogs	11
autumn	13	dig	65	soaked	11	hair	10
carrots	12	pit	20	chemical	10	sound	10
rains	10	digging	42	tnt	10	puppies	9
areas	10	buried	10	dangerous	9	lilies	9
children	10			shovel	8	protect	9
yesterday	10			gasoline	8	puppy	8
corn	10			gases	8	lathyris	8
				combat	8	vibration	7
				killing	7	chickens	6
				exterminate	7	imperialis	5
				attack	7	dung	6
				phosphide	6		

**Table 2:** Top terms per cluster.

Clusters	Top Terms Per Cluster
Cluster 0	mole, plant, garden
Cluster 1	hole, repellent, cat
Cluster 2	buried, reduce, blind

## Data Availability

The data presented in this study are available as Supplementary Files. These data were derived from resources available in the public domain presented in Appendix A, Table A1.

## Supplementary Materials

The following supporting information can be downloaded at: https://www.mdpi.com/article/10.3390/ani14243611/s1.

## Appendix A

Table A1 presents a selection of the online sources identified as viable for the corpus collection.

**Table A1:** The main online sources included in the dataset.

Selected Sources Links
Last accessed on 24 October 2024:
https://forum.softpedia.com/topic/728830-cum-scapam-de-cartite/
https://comunitate.desprecopii.com/forums/topic/72419-cum-sa-scap-de-cartite/
https://www.elforum.info/topic/132616-cum-scap-de-cartite/
https://www.reno.ro/Cartite-t299908.html
https://plant-shop.ro/blog/cum-scapam-de-cartitele-din-gradina-metode-bio-si-eco/
https://casasidesign.ro/cartita-excavatorul-gradinii.html
https://www.casadex.ro/2019/03/cum-scap-de-cartitele-din-gradina-metode-fara-otrava/
https://www.misiuneacasa.ro/forum/.../gradina-curte/29215-trebuie-sa-scap-de-cartite
https://www.facebook.com/IdeiPracticePentruCasa/posts/2295942657138237/
https://www.facebook.com/groups/doctorulflorilor/
https://www.facebook.com/groups/seminte.libere/
https://www.facebook.com/groups/capsunisicapsunari/
https://www.facebook.com/groups/1100531637105946/
https://www.facebook.com/groups/740526862768545/
https://www.facebook.com/groups/1394371967351828/
https://www.facebook.com/adrian.asoltanie/posts/
https://www.facebook.com/groups/884706728310679/permalink/6096076310507002/
https://www.facebook.com/nicolae.hulpoi/posts/
https://www.facebook.com/paradisverde.1/posts/
Last accessed on 1 September 2023:
https://egradini.ro/forum/threads/combaterea-cartitelor.2910/
